# Alteration in circulating metabolites during and after heat stress in the conscious rat: potential biomarkers of exposure and organ-specific injury

**DOI:** 10.1186/s12899-014-0014-0

**Published:** 2014-12-24

**Authors:** Danielle L Ippolito, John A Lewis, Chenggang Yu, Lisa R Leon, Jonathan D Stallings

**Affiliations:** The United States Army Center for Environmental Health Research, Environmental Health Program, Bldg. 568 Doughten Drive, Fort Detrick, Frederick, MD 21702-5010 USA; Biotechnology High Performance Computing Software Applications Institute, Frederick, MD 21702-5010 USA; Thermal Mountain Medicine Division, US Army Research Institute of Environmental Medicine, Natick, MA 01760-5007 USA

**Keywords:** Heat stress, Metabolomics, Systems biology, Energetics, Metabolic networks

## Abstract

**Background:**

Heat illness is a debilitating and potentially life-threatening condition. Limited data are available to identify individuals with heat illness at greatest risk for organ damage. We recently described the transcriptomic and proteomic responses to heat injury and recovery in multiple organs in an *in vivo* model of conscious rats heated to a maximum core temperature of 41.8°C (T_c,Max_). In this study, we examined changes in plasma metabolic networks at T_c,Max_, 24, or 48 hours after the heat stress stimulus.

**Results:**

Circulating metabolites were identified by gas chromatography/mass spectrometry and liquid chromatography/tandem mass spectrometry. Bioinformatics analysis of the metabolomic data corroborated proteomics and transcriptomics data in the tissue at the pathway level, supporting modulations in metabolic networks including cell death or catabolism (pyrimidine and purine degradation, acetylation, sulfation, redox alterations and glutathione metabolism, and the urea cycle/creatinine metabolism), energetics (stasis in glycolysis and tricarboxylic acid cycle, β-oxidation), cholesterol and nitric oxide metabolism, and bile acids. Hierarchical clustering identified 15 biochemicals that differentiated animals with histopathological evidence of cardiac injury at 48 hours from uninjured animals. The metabolic networks perturbed in the plasma corroborated the tissue proteomics and transcriptomics pathway data, supporting a model of irreversible cell death and decrements in energetics as key indicators of cardiac damage in response to heat stress.

**Conclusions:**

Integrating plasma metabolomics with tissue proteomics and transcriptomics supports a diagnostic approach to assessing individual susceptibility to organ injury and predicting recovery after heat stress.

**Electronic supplementary material:**

The online version of this article (doi:10.1186/s12899-014-0014-0) contains supplementary material, which is available to authorized users.

## Background

Heat illness and heat stroke, the most severe form of heat illness, are life-threatening conditions characterized by elevations in core temperature (T_c_) resulting from an inability to adequately dissipate excess body heat to the environment. The intrinsic nature of military operations (i.e., heavy physical activity in extreme environments) places military personnel at greater risk of developing heat-related illnesses. Sustained military operations in the Middle East have been accompanied by an increase in the number of heat stroke cases on the battlefield and in training. During the past two decades alone the incidence of heat stroke has increased over seven-fold [1]. Moreover, the 30-year mortality rates from heart, kidney, and liver failure in US forces increases by 40% in individuals with a history of heat stroke [2,3]. In 2013, US forces sustained over 2000 heat-related injuries requiring hospitalization, including 324 cases of heat stroke [1]. The actual incidence is projected to be considerably higher when considering undocumented instances that never reach triage [4,5]. In a five-year retrospective study, 10,319 cases of heat injury required medical resources to treat, including 1872 cases of heat stroke [4]. The estimated cost to the military approaches $52 million per year (USD), assuming a cost of $6200 per day, an average hospital stay of 3.2 days, and 2620 cases of heat injuries and heat stroke per year [6]. Other costs include duty days and salary lost during recovery (up to 5.5 months) and the loss in investment and training associated with service discharge for medical reasons [6]. Taken together, these statistics indicate that there is an urgent need for earlier indicators of organ injury and susceptibility and molecular based indicators to improve return to duty decisions [7].

The physiological responses to an excess heat load include elevations in heart rate, a drop in mean arterial pressure, attenuated sweating rates, stupor, and collapse. The molecular level alterations and influence of the physiological events preceding and contributing to the systemic inflammatory response associated with heat stroke remain largely unknown. Heat stroke compromises tight junction integrity in the gut, resulting in leakage of bacteria into the circulation [8]. If uncontrolled, the ensuing thermoregulatory and immune responses can progress to system inflammatory response syndrome (SIRS) and ultimately multi-organ failure and death [9-13]. Designing novel and effective treatment and detection strategies for heat stroke requires a better understanding of the physiological and molecular alterations that accompany heat stroke and characterize the SIRS event. However, in human studies 39.0 – 39.5°C is the highest ethically attainable maximal core temperature (T_c,Max_), but these temperatures are insufficient to induce heat stroke. To overcome these ethical limitations, we recently described conscious rat and mouse models of heat illness [10,11,14-18]. The models use abdominally implanted radiotelemetry units to track T_c_ while supporting physiological and behavioral adjustments to heat stress. In a recent study, we used our physiological model of heat stress to conduct an integrated systems biology evaluation of transcriptomic and proteomic changes in heart, liver, kidney, and lung after heat stress, heat injury, and recovery [19]. We identified discriminatory gene and protein signatures in heat-injured cardiac tissue reflecting perturbations in oxidative phosphorylation, energy production, and inflammatory response.

These global changes in metabolic networks associated with energy production in target tissues suggest that more accessible biofluids (e.g., serum, plasma, urine, and saliva) are also likely to reflect changes in the physiological state of the heat-stressed and heat-injured organism. Metabolomic profiling of plasma in conjunction with proteomic and transcriptomic analysis has recently emerged as a powerful predictive tool reflecting the dynamic responses to genetic modification and physiological, pathophysioloigcal, and/or developmental stimuli. However, perturbation in metabolic networks has not been well studied in heat illness. Understanding the metabolic response to heat illness and subsequent organ injury provides unique insight into understanding how mammalian systems react to heat illness and recovery. Further, methods to interrogate metabolites in accessible biofluids have been developed to allow a global assessment of organism response to environmental stressors [20]. One disadvantage of metabolomics is the likelihood of false positives given the metabolome’s exquisite sensitivity to subtle changes in physiology (e.g., food intake, changes in temperature, stress, etc.). Therefore, any study of the metabolome must discriminate pathological changes in metabolic networks from changes inherent in normal physiological functioning. Integrating metabolic networks at the level of molecular function is one approach to differentiate changes related to heat-stress and/or heat-injury from physiological variation in the unperturbed system. Bioinformatics methods can be used to identify an integrated panel of multiple physiological networks and biomarkers.

In this study, we evaluate the metabolomic profile in response to heat stress and heat injury, and compare the results with the proteomic and transcriptomic profiles described previously. We demonstrate plasma metabolomics profiles unique to the physiological conditions of heat stress, heat injury in cardiac tissue, and heat stress without injury. Metabolomics network analysis demonstrates perturbations in biological processes associated with energy usage and cell death, similar to the transcriptomics and proteomics analyses conducted previously. Further, integrated analysis of variance (ANOVA) analysis, random forest analysis, and hierarchical clustering analysis identify panels of biochemicals differentiating controls from heat-stressed and heat-injured animals.

## Results and discussion

### Thermoregulation and histopathology

T_c,Max_ was reached at 2–3 hours, as previously published by our laboratories in more detailed thermoregulation analyses conducted with these animals and other similar studies [19,21]. Animals in the 24 and 48 hour cohorts were allowed to recover for the specified time prior to termination. In thermoregulation studies with these animals, temperatures return to baseline by 24–48 hours, but cardiac-injured animals exhibit hypothermia before recovery, as published in detail by our laboratory [19].

Liver, kidney, and heart were assessed by histopathology and scored for evidence of injury as described previously [19]. The heart was the only tissue with treatment-related evidence for injury, with inflammation and cardiomyopathy in three out of six animals in the heated cohort as described in detail in a recent publication from our laboratory [19] (Additional file 1A). For the remainder of the study, animals in the 48 hour treatment group were sub-stratified into cohorts according to uninjured vs. injured.

### The metabolomics heat stress response and recovery in plasma—biochemical networks

#### Overview of metabolic network changes

**Figure 1:**
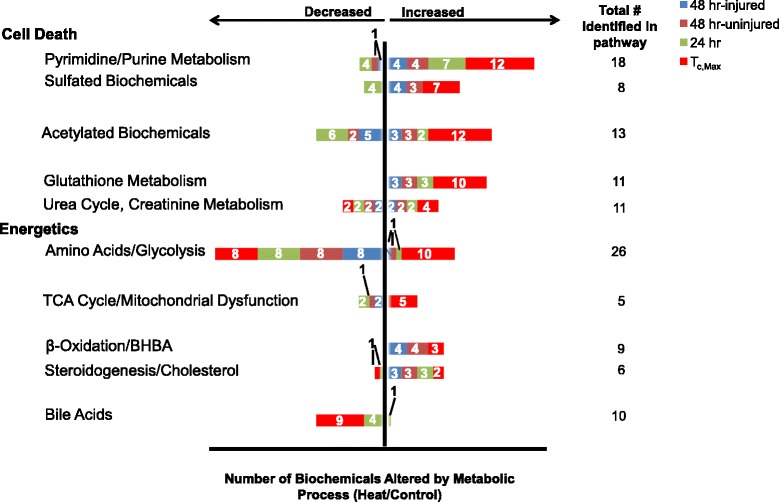
**Altered metabolic pathways and process networks in plasma of rats after heat stress.** Biochemicals were grouped by KEGG super-pathway and sub-pathway. Pathways meeting significance thresholds were plotted by number of affected metabolites per total number identified in the plasma (at right). Significance thresholds were determined by ANOVA, by ratio of heat/control at T_c,Max_, 24 or 48 hours. The 48 hour endpoint was further subdivided into injured or uninjured animals by cardiac histopathology.

A total of 422 biochemicals were identified that matched 307 named/identified chemicals in Metabolon’s reference library, with 115 unidentified biochemicals (Additional file 1). The largest change in number of biochemicals relative to controls occurred at T_c,Max_, with fewer alterations at 24 and 48 hours. The key biochemicals represented pathways related to the following categories: cell death or catabolism (pyrimidine and purine degradation, acetylation, sulfation, redox alterations and glutathione metabolism, and the urea cycle/creatinine metabolism), energetics (stasis in glycolysis and tricarboxylic acid [TCA] cycle, β-oxidation), cholesterol and nitric oxide metabolism, and bile acids (Figure 1).

T_c,Max_ showed the greatest percentage of biochemicals altered in response to heat injury in virtually all categories. At T_c,Max_, nearly 100% of identified biochemicals associated with cell death in a given pathway were elevated, suggesting an overall increase in biosynthesis to combat an overt exposure to redox stress (Figure 1). Biochemicals in all enriched metabolic pathways relating to cell death were up-regulated at T_c,Max_, with progressively fewer biochemicals within each pathway elevated and/or reverting to down-regulation over time (Figure 1).

In a two-way ANOVA with contrasts evaluating changes across all time points, seven key biomarkers demarcated heat stress from control during recovery: 5,6-dihydrouracil, 3-ureidopropionate, GSSG, ornithine, creatinine, corticosterone and pyridoxal (Table 1).

**Table 1:** List of seven key biochemical differentially expressed in heat-stressed and control animals*

**Identified biochemical (pathway)**	**Literature review (biochemical and/or pathway)**	**References**
5,6-Dihydrouracil (pyrimidine metabolism)	Early signal of apoptosis; DNA damage from reactive oxygen species elevation; deficiency	[22-25]
3-Ureidopropionate (alanine/aspartate metabolism)	Increased reactive oxygen species; inhibition of mitochondrial energy metabolism; neurotoxic/excitotoxic	[26]
Ornithine (urea cycle, arginine metabolism)	Production of arginine and increase in autophagy, cell death, removal of excess NH4^+^, energy metabolism; slight renal dysfunction	[27]
Glutathione disulfide, oxidized (redox)	Apoptosis, DNA damage, cell proliferation, survival, differentiation, metabolism; redox stress and/or crisis due to elevated reactive oxygen species	[28-30]
Corticosterone (steroid/sterol metabolism)	Mitochondrial conversion of acetyl-CoA to cholesterol and conversion of cholesterol to corticosterone; adrenal cortex production of cholesterol to pregnenalone, and ultimately cortisol; involved in glucocorticoid activity and stress response	[31]
7-α-Hydroxy-3-oxo-4-cholestenoate [7-HOCA] (steroid/sterol metabolism)	Bile acid synthesis from cholesterol; CYP7A1 activity in the liver; bile acid synthesis	[32]
Pyridoxal (vitamin B metabolism)	decreased renal function; Anabolism; cofactor for reaction releasing glucose from glycogen	[33]

*Not segregated based on cardiac injury.

### Redox stress and cell death

**Figure 2:**
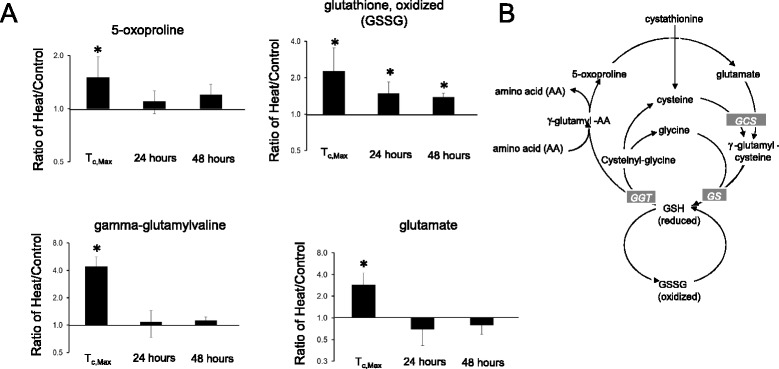
**Initial elevation in glutathione metabolism with recovery by 24–48 hours. (A)** Biochemicals altered in heat exposure and disposition after recovery (fold-change, heat/control). **(B)** The glutathione metabolic pathway. Circled biochemicals represent changes observed after heat exposure. *P < 0.05 heat exposed versus control rat, two-way ANOVA with contrasts. AA, amino acid; GCS, γ-glutamyl cysteine synthase; GGT, γ-glutamyl transferase; GS, glutathione synthase.

The glutathione (GSH) metabolic network exhibited profound perturbation in response to heat stress (Figure 2). All three biochemicals involved in glutathione metabolism were elevated at T_c,Max_, probably as a result of heat-initiated redox crisis (Figure 2). γ-Glutamylated amino acids (including alanine, glutamine, isoleucine, leucine, phenylalanine, tyrosine, and valine [Additional file 1]), 5-oxoproline, cysteine-GSH disulfide, and glutamate were significantly greater than control levels at T_c,Max_. γ-Glutamylmethionine was slightly elevated at T_c,Max_, but the difference did not reach statistical significance (Additional file 1). At 24 and 48 hours, GSSG remained elevated. At 48 hours, three of the 11 biochemicals in GSH redox regulation remained elevated (γ-glutamylphenylalanine, 5-oxoproline, and oxidized glutathione [GSSG]).

Production of reactive oxygen species (ROS) and oxidative stress occur when the cell’s protective mechanisms are saturated. ROS also play key signaling roles in cell proliferation, survival, disease and pathophysiology [28]. Increased ROS may lead to apoptosis, DNA damage, cell proliferation, survival, differentiation, and disruption in metabolism [34]. Heat exposure elevates free radicals [29]. Free radicals and other ROS are neutralized by GSH in redox regulation cycles. Deficient and/or dysfunctional GSH can disrupt cell processes and lead to cell death [30]. Oxidized GSH can be reduced back by glutathione reductase with NADPH (nicotinamide adenine dinucleotide phosphate) as an electron donor. GSH donates a reducing equivalent to unstable redox species. Under normal physiological conditions, more than 90% of the glutathione pool is in the GSH form and less than 10% is in the disulfide GSSG form. The ratio of GSH to GSSG is a metric of cellular toxicity, with elevated GSSG indicating oxidative stress [30]. Taken together, these results suggest that early perturbations in metabolic networks elicit persistent redox stress out to 48 hours after exposure. These persistent metabolic changes in heat-stressed individuals may affect performance during subsequent episodes of heat injury.

### Arginine metabolism, nitric oxide metabolism, and cell death

**Figure 3:**
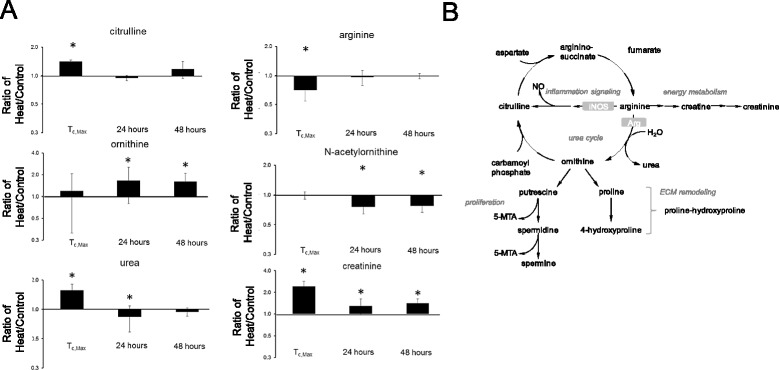
**Alterations in arginine metabolism and the urea cycle after heat exposure. (A)** Change in plasma arginine metabolism biochemicals as a function of time after heat exposure (fold-change, heat/control). **(B)** Biochemical pathway of inflammation signaling, the urea cycle, and energy metabolism. *p < 0.05 heat exposed versus control rat, two-way ANOVA with contrasts.

Six biochemicals involved in arginine metabolism were altered after heat exposure (Figure 3). These biochemicals were involved in the urea cycle, which feeds creatine-to-creatinine production during metabolic mobilization of the muscle and/or brain energy reserves during energy crisis. Heat stress increased citrulline, decreased arginine, and increased urea in rat plasma at T_c,Max_ (Figure 3A). Trans-4-hydroxyproline was not significantly changed in concentration after heat stress (Additional file 1).

Elevated citrulline levels suggest a concomitant increase in nitric oxide, a product of inflammatory signaling (Figure 3B) [27]. Increased urea may be associated with cell death, autophagy, and/or protein degradation. The combination of elevated urea and creatinine could also suggest renal dysfunction, consistent with the proteinosis observed in the renal histopathology as reported in the companion study. Elevated plasma creatinine at later time points could indicate continued use of skeletal muscle energy reserves and persistent renal dysfunction. Concomitantly decreased arginine and increased urea may indicate slower kinetics of arginine metabolism with increased urea production (Figure 3B). Increased urea may also be the result of an increase in NH_4_^+^, a byproduct of cellular death [35].

At 24 and 48 hours, citrulline levels were no longer elevated, suggesting that nitric oxide (NO) production had decreased. Interestingly, however, ornithine levels increased at 24 and 48 hours, possibly reflecting slower arginine metabolism kinetics in general [36]. Taken together, these data suggest that a slight renal dysfunction was maintained during recovery, consistent with histopathologic evidence suggesting renal dysfunction.

### Purine/Pyrimidine metabolism and cell death

Fourteen out of 18 identified biochemicals in the pyrimidine and purine metabolic pathways were significantly different at T_c,Max_ relative to control (Figure 1 and Additional file 2). Exceptions were adenosine monophosphate (AMP), uridine, cytosine, and dihydroorotate. Uracil, 5,6-dihydrouracil, 3-ureidopropionate, β-alanine, and an acetylated form of β-alanine, N-acetyl-β-alanine, were all significantly increased at T_c,Max_ compared to control rats, suggesting that a significant proportion of biochemicals involved in pyrimidine degradation was altered (Additional file 2). Significantly, nearly all the biochemicals immediately upstream of β-alanine metabolism in the pyrimidine metabolism reference Kyoto Encyclopedia of Genes and Genomes (KEGG) pathway are modulated, suggesting that a significant proportion of this biochemical network is affected by heat stress at T_c,Max_. In plant cells, alterations in pyrimidine nucleotide metabolism are considered an early signal of apoptosis and often are induced by an increase in endogenous NO [22]. 3-ureidopropionate, the substrate of the enzyme dihydropyrimidinase, was elevated (Additional file 1) while the product, 5,6-dihydrouracil, was decreased (Additional file 2), suggesting a down-regulation in the level of dihydropyriminidase activity or 3-ureidopropionate. Further, carnosine is synthesized from L-histidine and β-alanine, with β-alanine as the rate-limiting precursor [37], and 3-ureidopropionate as the precursor to β-alanine production. The result of these alterations in pyrimidine degradation could suggest DNA damage by endogenous ROS over-riding cellular repair and protection mechanisms. Several altered biochemical pathways support elevated ROS.

### Acetylation or sulfation and cell death

Of the N-acetylated biochemicals detected, 14 of 15 compounds were significantly elevated at T_c,Max_ (Additional file 3). While 8 of 15 biochemicals were significantly changed at 24 hours, 6 remained altered at 48 hours. Heat exposure appeared to change in the activity of acetyltransferases or deacetylases, consistent with cellular damage. This initial response probably reflects hypoxia and initiation of cell death [38]. Acetyltransferases and deacetylases may allow an organism to adapt to recovery.

Eight sulfated compounds were detected and at T_c,Max_, of which seven were significantly increased (Additional file 4). In addition, seven unnamed, putatively sulfated compounds also increased at T_c,Max_ (X-12182, X-12183, X-12184, X-12185, X-12230 and X-12307; Additional file 1). At 24 hours, all biochemicals which had been higher than control at T_c,Max_ were now significantly lower than controls, with a reversal of the trend after 48 hours (i.e., higher than controls; Additional file 4). Sulfotransferase enzymatic activity may have been induced or the activities of sulfatases may have reduced initially to facilitate clearance of cellular debris [35], then returned to control levels after recovery.

### Amino acids and cell death

Of the 19 identified amino acids, seven were decreased and eight were increased relative to controls at T_c,Max_, with a trend toward overall down-regulation at 24 hours and 48 hours. The exception was lysine, with a trend toward an increase at T_c,Max_, with elevations at both 24 and 48 hours (Additional file 5). At T_c,Max_, serine, threonine and alanine were lower than control. All three amino acids are synthesized from glycolytic intermediates. Although not definitive, these results may suggest a decrease in glycolysis [35]. Moreover, glutamate, which is consumed in the production of these amino acids, was increased at T_c,Max_. Whether these amino acid changes are indicative of energetic alterations or increased autophagy is unclear [39]. However, it is interesting to note that, at T_c,Max_, amino acids used as anaplerotic contributors to the TCA cycle (i.e., glutamate and the branched-chain amino acids leucine, isoleucine, and valine) were increased and amino acids potentially synthesized as a consequence of glycolysis were decreased (serine, alanine, and threonine) (Additional file 5). These results may indicate perturbations in glycolysis and an overall slowing of energy production.

### TCA cycle intermediates and energetics

**Figure 4:**
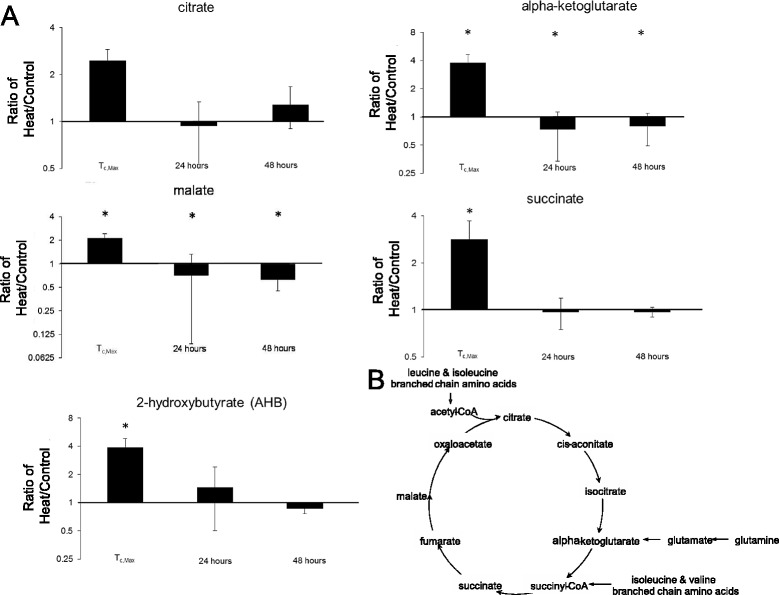
**Mitochondrial dysfunction suggested by elevated TCA cycle intermediates and 2-hydroxybutyrate (AHB) at T**_**c,Max**_ **. (A)** Fold-change from control in biochemicals implicated in the TCA cycle. **(B)** The TCA cycle, with red circles indicating biochemicals with significant deviation from control after heat stress. *p < 0.05 heat exposed versus control rat, two-way ANOVA with contrasts.

All five of the TCA cycle intermediates identified at T_c,Max_ were higher than controls (Figure 4), suggesting mitochondrial dysfunction and energy crisis. The concomitant increase in 2-hydroxybutyrate (AHB) further supported a perturbation in mitochondrial function [35]. By 24–48 hours, TCA metabolites in the plasma were no longer significantly elevated, suggesting restoration of the TCA cycle to homeostasis.

### β-Oxidation, cholesterol synthesis, and bile acids

3-Hydroxybutyrate (BHBA) and carnitines were significantly increased at T_c,Max_ (Additional file 6), supporting an increase in fatty acid β-oxidation for energy production. Increased β-oxidation is also implied from a trend toward lower plasma levels of the medium-chain fatty acids (e.g., palmitoylcarnitine, stearoylcarnitine, and oleoylcarnitine) relative to controls. These fatty acids can enter the mitochondria without requiring transporter activity (Additional file 6). Further, hexanoylcarnitine, a long-chain fatty acid conjugated to carnitine to facilitate entry into the mitochondria [35], is increased in heated animals. Increased β-oxidation may increase levels of acetyl coenzyme A (acetyl CoA) (Additional file 1, Additional file 6A), but the profound down-regulation in glycolysis, TCA cycle, and amino acid metabolism suggests that the acetyl CoA is not being used for adenosine triphosphate (ATP) production at T_c,Max_.

β-Oxidation may also be up-regulated to facilitate cholesterol synthesis and corticosterone production [35]. At T_c,Max_, corticosterone was significantly increased in heated animals relative to controls, and the increase persisted to 24–48 hours (Additional file 6). The increase in BHBA may reflect an increase in acetyl CoA for subsequent cholesterol metabolism and steroidogenesis, but functional mitochondria are required for this process. Alternatively, the elevated corticosterone could be released from the adrenal cortex during heat stress as part of a generalized stress response. Studies suggest prophylactic treatment with glucocorticoids protects against heat stress [40]. Changes in corticosterone levels could gauge severity of perturbations during heat and recovery [31].

Bile acids and steroids are generated from cholesterol. Following heat stress, plasma levels of steroids increased with a concomitant decrease in bile acids, suggesting *de novo* steroidogenesis (Additional file 7). Bile acid concentration is usually low in the normal systemic circulation. With heat stress, levels of nearly all bile acids detected were even lower at T_c,Max_ (Additional file 7), possibly due to decreased reuptake within the small intestine and constricted intestinal circulation [41].

### Co-factor metabolism

In addition to the broad categories of energetics and cell death, metabolites of Vitamin B_6_ are altered by heat stress (Additional file 8). Heat stress resulted in an accumulation of pyridoxate (a breakdown product secreted in the urine), potentially reflecting renal dysfunction. Pyridoxal levels were increased in heated animals relative to controls at T_c,Max_, possibly due to increased catabolism. In contrast, pyridoxal and pyridoxate levels were lower than control at 24–48 hours, possibly due to increased anabolism (a process that requires B_6_ as a cofactor) and return to homeostasis (Additional file 8).

### Data integration—random forest analysis to identify biochemicals which discriminate heat exposure from unheated controls

**Figure 5:**
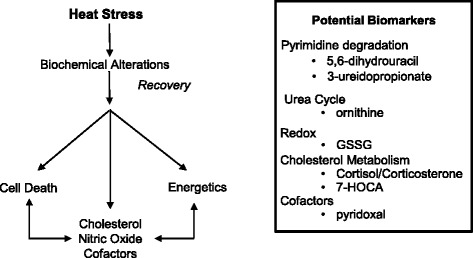
**Model of heat stress and recovery and potential biomarkers.** Hierarchical clustering was used to compare heated and control animals 48 hours after heat exposure. Data clustered by cardioinflammation score in animals with the greatest histopathological evidence of injury.

Taken together, the results of the metabolic pathway analysis supports a model of heat stress perturbing metabolic networks affecting cell death and energetics. Ultimately, these metabolic network perturbations lead to disruption of cholesterol and bile acid synthesis, nitric oxide production and inflammatory signaling, and vitamin B_6_ cofactor regulation (Figure 5). Performing integrated ANOVA analysis across biochemicals at all time points identified seven biomarkers within five metabolic networks which discriminated heat stressed individuals from controls at any time point (Figure 5).

**Figure 6:**
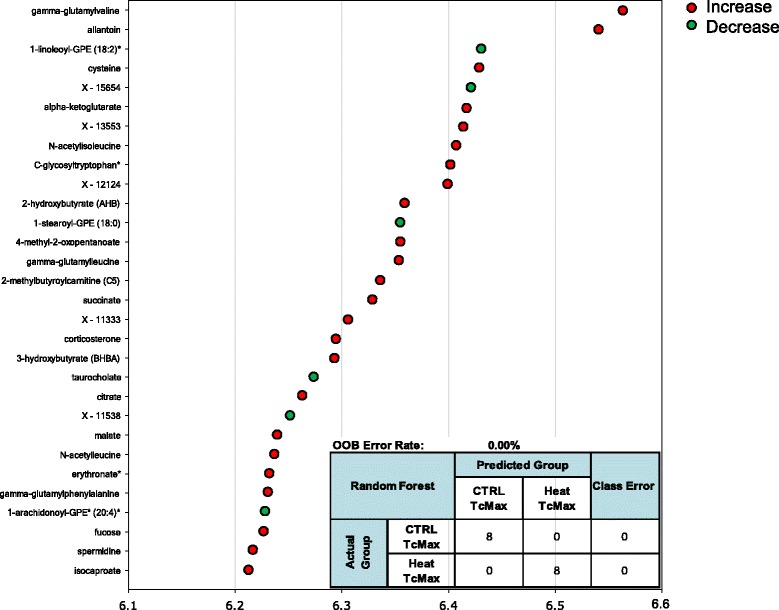
**Random forest analysis accurately discriminates heat exposure from unheated controls at T**_**c,Max**_ **.** The top 30 metabolites were identified in out-of-bag (OOB) selection to have 100% predictive power by random forest analysis at T_c,Max_. The inset represents predictive power of the 30 biochemicals identified. Colored circles indicate biochemicals that were also significantly up (red) or down (green) regulated, p < 0.05, 2-way ANOVA with contrasts. Biochemicals marked by an asterisk indicate likely identifications based on MS/MS fragmentation and other chemical properties.

**Figure 7:**
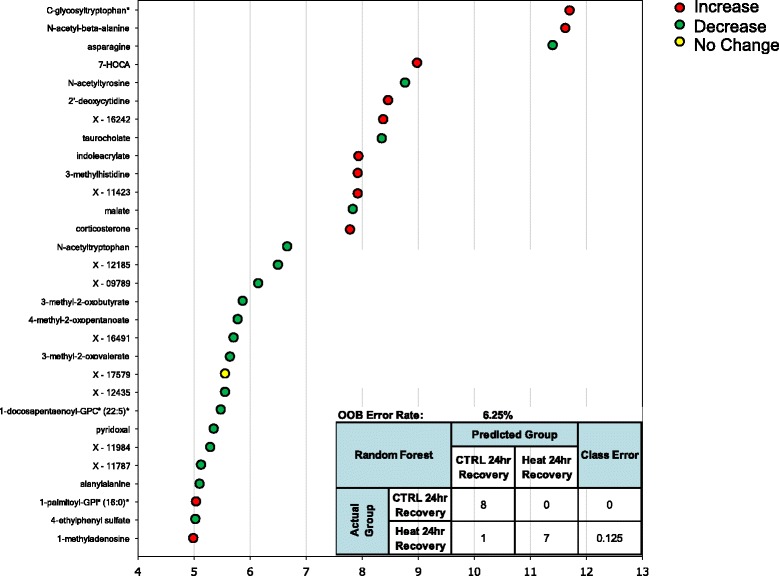
**Random forest analysis accurately discriminates heat exposure from unheated controls at 24 hours.** The top 30 metabolites were identified in OOB selection to have 94% predictive power by random forest analysis at T_c,Max_. The inset represents predictive power of the 30 biochemicals identified. Colored circles indicate biochemicals that were also significantly up (red) or down (green) regulated, p < 0.05, 2-way ANOVA with contrasts. One biochemical was not significantly altered by ANOVA analysis (yellow). Biochemicals marked by an asterisk indicate likely identifications based on MS/MS fragmentation and other chemical properties.

**Figure 8:**
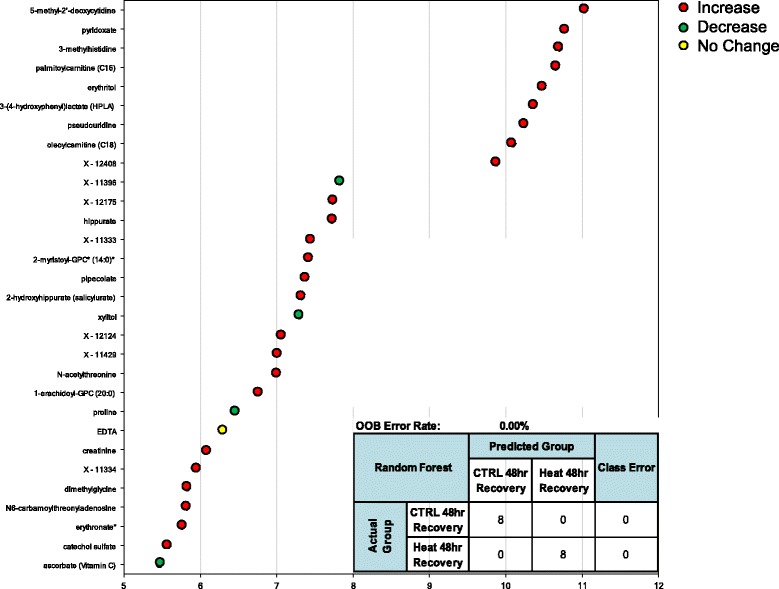
**Random forest analysis accurately discriminates heat exposure from unheated controls at 48 hours.** The top 30 metabolites were identified in OOB selection to have 100% predictive power by random forest analysis at 48 hours. The inset represents the top-scoring molecule (5-methyl-2’deoxycytidne). Colored circles indicate biochemicals that were also significantly up (red) or down (green) regulated, p < 0.05, 2-way ANOVA with contrasts. One biochemical was not significantly altered by ANOVA analysis (yellow). Biochemicals marked by an asterisk indicate likely identifications based on MS/MS fragmentation and other chemical properties.

Random forest statistical analyses were used to determine sets of biomarkers capable of discriminating control from heat stress at T_c,Max_ (Figure 6), 24 hours (Figure 7) and 48 hours (Figure 8) after exposure to heat. The out-of-bag (OOB) error rate for each forest plot was 0%, 6.25%, and 0%, respectively. The top predictive chemicals at T_c,Max_ were γ-glutamylvaline and allantoin (Figure 6). Allantoin is not typically found in humans, but γ-glutamylvaline is elevated in response to perturbations in the redox cycle [28] (see also Figure 2). At 24 hours, the top predictive chemicals were C-glycosyltryptophan, N-acetyl-β-alanine, and asparagine (Figure 7). The predictive power of the model was slightly lower than T_c,Max_, but still predicted with nearly 94% accuracy. At 48 hours, the most predictive biochemicals were 5-methyl-2’deoxycytidine, pyridoxate, 3-methylhistidine, palmitoylcarnitine (C16), erythritol, 3-(4-hydroxphenyl) lactate (HPLA), pseudouridine, oleoylcarnitine (C18), and X-12408 (Figure 8). The degradation product of DNA (5-methyl-2’-deoxycytidine) and pseudouridine (a representative of RNA degradation) are both likely representative of continued cell death (see also Additional file 2) [22].

### Identification of predictive indicators of persistent cardiac injury at 48 hours

**Figure 9:**
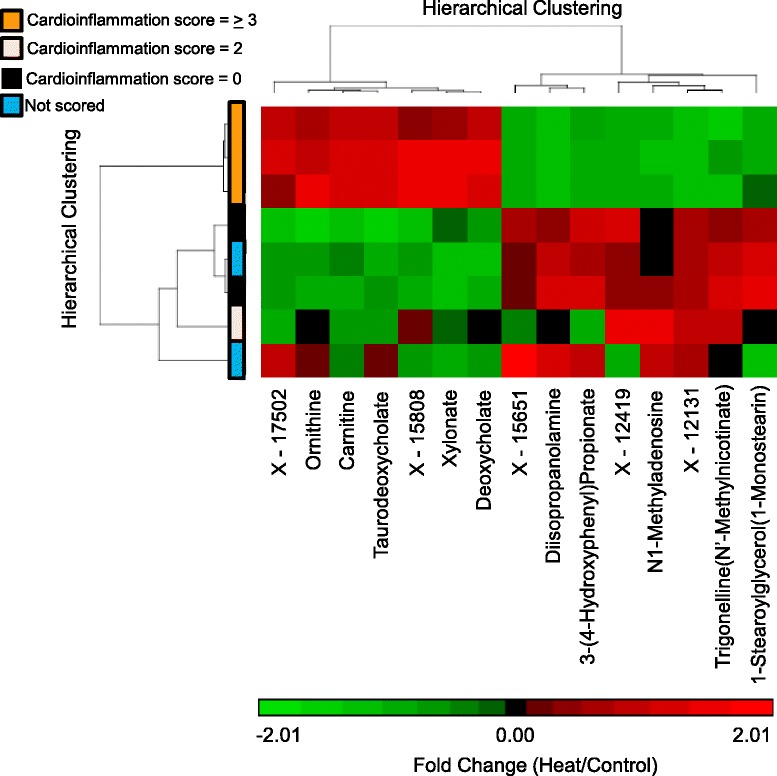
**Metabolites discriminate animals with histopathologic evidence of heart injury versus uninjured animals at 48 hours.** Hierarchical clustering was used to compare heated and control animals 48 hours after heat exposure. Data clustered by cardioinflammation score in animals with the greatest histopathological evidence of injury.

At 48 hours, three out of six heated animals showed histopathological evidence of cardiac injury (see companion study). Segregating injured from uninjured animals did not result in significant differences at 48 hours for any biochemical pathway grouping except acetylation and sulfation (Additional files 1, 3, and 4). Using hierarchical clustering at 48 hours, we evaluated the global change in metabolites after heat exposure, segregating the 48 hour animals into injured and uninjured cohorts (n = 3 per cohort). A panel of 15 biochemicals discriminated animals with histopathologic evidence of heart injury from controls and uninjured, heated animals (Figure 9). Biochemicals in the panel were sub-categorized by metabolic pathway (Table 2). Ornithine and N1-methyladenosine and taurodeoxycholate have been associated with cardiac injury (summarized in Table 2).

**Table 2:** **Review of 15 metabolites differentiating animals with histopathological evidence of cardioinflammation from uninjured animals**

**Identified biochemical (pathway)**	**Literature review (biochemical and/or pathway)**	**References**
Ornithine (phenylalanine and tyrosine metabolism)	inhibition of nitric oxide; relationship between nitric oxide modulation of the Frank-Starling response in heart; nitric oxide and nitric oxide synthase are sensitive to thermal stress in fish	[42]
3-(4-Hydroxyphenyl) propionate (phenylalanine and tyrosine metabolism)	Biological nitrification inhibition (in plants); phenylalanine and tyrosine concentrations are reduced after Hsp70 increase and heat stress (in yeast)	[43,44]
N1-Methyladenosine (purine metabolism, adenine containing)	N1-methyladenosine analogues are cardioprotective agents in ischemic reperfusion model; decreased infarction; purine metabolism associated with myocardial steatosis and down-regulation of adipose triglyceride In heart	[45,46]
Xylonate (nucleotide sugars, pentose metabolism)	Deficiency in pentose metabolism produces a protective effect through decreased cholesterol synthesis, superoxide production, and reductive stress	[47]
1-Stearoylglycerol (1-Monostearin) (monoacylglycerol)	Associated with increased lipid catabolism and remodeling mitochondrial oxidation to aerobic glycolysis (hepatocellular carcinoma)	[48]
Carnitine (carnitine metabolism)	Disrupted carnitine metabolism is associated with mitochondrial dysfunction and increased pulmonary flow (lamb model); cardioprotective by increasing heat shock protein synthesis in adriamycin-induced cardiomyopathy	[49-51]
Taurodeoxycholate (bile acid metabolism)	Bile acids exert a protective effect after ischemic injury in porcine hearts; cause endoplasmic reticulum mitochondrial stress; deoxycholate and taurodeoxycholate affect heart mitochondria by decreasing respiration, affecting membrane potential, inducing mitochondrial permeability transition, and altering mitochondrial bioenergetics; impaired cardiac mitochondrial function may cause cardiac alterations in cholestasis	[52-55]
Deoxycholate (bile acid metabolism)	(see above)	(see above)
Trigonelline (N’-Methylnicotinate) (nicotinate and nicotinamide metabolism)	Cardioprotective effects after isoproterenol induced myocardial dysfunction (reduction in Hsp27, αB-crystallin and calcium/calmodulin dependent kinase-II-δ)	[56]
Diisopropanolamine (xenobiotics - chemical metabolism)	Increases choline uptake without affecting phospholipid synthesis (Chinese hamster ovary cells)	[57]
X – 17502 (Unknown)	n/a	n/a
X – 12419 (unknown)	n/a	n/a
X – 15808 (unknown)	n/a	n/a
X – 12131 (unknown)	n/a	n/a
X – 15651 (unknown)	n/a	n/a

The results of the random forest analyses at each time point were most concordant with the two-way ANOVA analysis at 24 hours (two of the seven biomarkers identified in both analyses—7-HOCA and corticosterone). At T_c,Max_, only corticosterone was common to both analyses, and at 48 hours, only pyroxidate was common to both analyses. It is important to note, however, that 88 of the 90 biochemicals that were excellent classifiers of exposure were also significantly altered according to the ANOVA analyses (as indicated by the red and green circles in Figures 6, 7, 8). Thus, rather than the manual and somewhat arbitrary selection of the top seven biochemical based on circulating levels, the random forest analysis pulls out a list of chemicals that in combination (whether up- or down-regulated) provide greater discriminatory power than a single biomarker, which may be a more powerful approach in populations that demonstrate significant individual variability in the metabolic response across time points.

### Comparison of metabolic networks enriched in heat injury with complementary proteomics and transcriptomics pathway analysis

**Figure 10:**
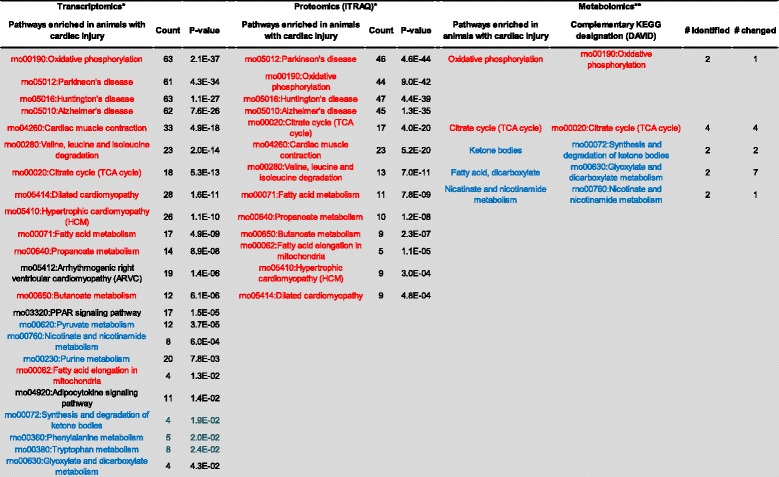
**Down-regulated biomarkers predictive of cardiac injury.** KEGG pathways negatively enriched in animals with cardiac injury were compared across transcriptomics, proteomics, and metabolomics studies in cardiac tissue. For metabolomics results, the Metabolon pathway designation is listed along with the complementary KEGG designation (in DAVID). The metabolomics studies are described in this study and the proteomics and transcriptomics results were described in detail in a previous study. *, previous study; **, this study; red text, common to at least two data sets, with the same direction in all data sets (up-regulated or down-regulated); blue text, common to at least two data sets, but direction (up-regulated or down-regulated) differs.

**Figure 11:**
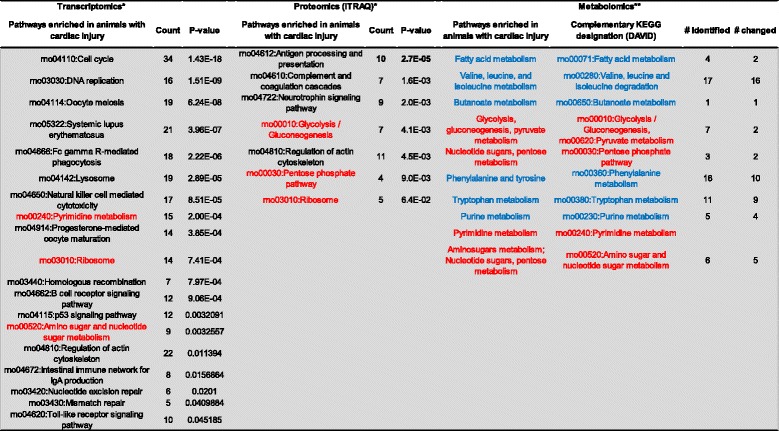
**Up-regulated biomarkers predictive of cardiac injury.** KEGG pathways positively enriched in animals with cardiac injury were compared across transcriptomics, proteomics, and metabolomics studies in cardiac tissue. For metabolomics results, the Metabolon pathway designation is listed along with the complementary KEGG designation (in DAVID). The metabolomics studies are described in this study and the proteomics and transcriptomics results were described in detail in a previous study. *, previous study; **, this study; red text, common to at least two data sets, with the same direction in all data sets (up-regulated or down-regulated); blue text, common to at least two data sets, but direction (up-regulated or down-regulated) differs.

The strength of metabolomics is in its analysis of hundreds of analytes simultaneously. Multiple small changes (<2-fold) within a given biochemical pathway can signal a physiologically relevant perturbation of a physiological network regulating a metabolic function [58]. These physiologically relevant changes may be overlooked in the analysis of single analytes. The network-wide comparison of plasma metabolites complements the transcriptomic and proteomic profiles observed in cardiac tissue in our recently published study [19]. The greatest concordance was observed in down-regulated biomolecules among KEGG pathways enriched in the three data sets (Figure 10). Metabolomics, proteomics, and transcriptomics data integration suggests concurrence in the transcripts, proteins, and metabolites supporting a down-regulation of oxidative phosphorylation and the TCA cycle. Taken together, these results support energy crisis and oxidative stress at 48 hours in cardiac-injured animals. Importantly, the metabolomics data obtained in plasma are concordant with tissue-based proteomics and transcriptomics profiles at the pathway level. Sampling blood (an accessible biofluid) indicates cardiac distress at the tissue level in this study. The metabolomics data were more discordant than the proteomics and the transcriptomics data, especially when comparing up-regulated genes and proteins with metabolites in the corresponding KEGG pathways. However, amino acid/nucleotide sugar metabolism were modulated at the transcript, protein, and metabolite level, supporting a stasis of energy production/energy crisis (Figure 11). Combined with the panel of potential cardiac injury markers (Figure 9), these results suggest a plasma-based series of metabolic indicators for tissue damage induced by heat stress. Detecting biomarkers predictive of cardiac injury in an accessible biofluid supports further studies investigating whether these metabolites represent viable biomarker candidates for predicting cardiac injury and recovery.

## Conclusion

In this work, we present a plasma metabolomic profile of heat stress and heat injury in an *in vivo* rodent model of heat stress. The metabolomic profile in plasma is concordant with tissue proteomics and transcriptomic profiles indicating energy crisis and oxidative stress, suggesting that metabolic indicators in the plasma may provide surrogate markers for tissue injury in an accessible biofluid. Coupled with the earlier work in tissue transcriptomics and proteomics in the heat-stressed rodent model [19], the global metabolomic profiles identified the basis for future work for modeling the response to heat stress and heat injury [18]. Integration of metabolomics, proteomics, and transcriptomics in a top-down manner will provide the foundation for network analysis and computational based experiments in a human 3D thermoregulation model [59]. Anchoring these systemic stress responses to the physiological model of heat stroke will provide further insight into forecasting risk of disease, timing of disease onset, and intensity of disease at the organ level. As a result, we anticipate that such a model would accelerate the development of tools to improve disease prevention, classification, and ultimately treatment.

## Methods

### Materials and methods

#### Animal model

*In vivo* rat experiments were performed at the US Army Research Institute of Environmental Medicine (USARIEM). The Institutional Animal Care and Use Committee approved all experimental procedures, which were performed in accordance with the American Physiological Society’s guiding principles for research involving animals and adhered to the high standard (best practice) of veterinary care as stipulated in the Guide for Care and Use of Laboratory Animals. Research was conducted in compliance with the Animal Welfare Act, and other Federal statutes and regulations relating to animals and experiments involving animals and adheres to principles stated in the “Guide for Care and Use of Laboratory Animals” (NRC 2011) as prepared by the Committee on Care and Use of Laboratory Animals of the Institute of Laboratory Animal Resources, National Research Council in facilities that are fully accredited by the Association for Assessment and Accreditation of Laboratory Animal Care, International. As previously described [18], male Fischer 344 (F344; n = 48; Charles River Laboratories, Stone Ridge, NY) rats weighing 234–336 g (~2–3 months old) were used. Briefly, rats were housed under standard laboratory conditions (22°C, 12:12 hour light:dark cycle, lights on at 6:00 AM) in an Association for Assessment and Accreditation of Laboratory Animal Care-accredited facility. A relatively cool housing temperature was chosen to support survival during heat-stress recovery [18], during which chow (Harlan Teklad, LM-485; Madison, WI) and water were provided *ad libitum* [60]. Rats were implanted with TL11M2-C50-PXT PhysioTel® Multiplus Transmitters (Data Sciences International, St. Paul, MN) to measure core temperature (T_c_; ±0.25°C), heart rate, and mean arterial pressure (±3 mmHg). Physiological response and temperature regulation after heat stress has been published in recent studies by our laboratories [18,21,59].

All experiments were conducted in conscious, free-moving animals, as previously described [18]. Briefly, rats were placed in a floor-standing incubator (Thermo Scientific, Ashville, NC) set at room temperature (RT, 22°C) 24 hours prior to initiation of heat stress experiments. Non-heated rats were not introduced to the incubator environment. Heat-stress experimentation was initiated after T_c_ of the control and experimental rats reached values of approximately 37.3°C. The following day, food and water were removed from the animal cages; the incubator temperature was increased to 37.0 ± 0.2°C; and the rats were heated until a T_c_ of 41.8°C (T_c,Max_) was reached, at which time they were removed from the incubator, weighed, placed in a new cage, and returned to normal housing temperature (22.0 ± 0.2°C). Animals in the 24 and 48 hour cohorts were allowed to recover for the specified time prior to termination. Time-matched control rats experienced the same experimental procedures as the heat-stressed rats, but remained at the normal housing temperature of 22.0°C throughout experimentation. Control and experimental animals were provided food and water ad libitum throughout recovery. Plasma, heart, liver, lung, and kidney were harvested at T_c,Max_, 24, or 48 hours with T_c_, mean arterial pressure, and heart rate monitored continuously throughout recovery.

#### Histopathology

At necropsy, tissues (heart, liver, lung, kidney) were fixed in paraformaldehyde, mounted, sectioned, and stained with hematoxylin and eosin (IHC World, Woodstock, MD). Twenty serial sections were cut per tissue, with three to five sections per slide. Inflammatory or degenerative lesions were graded on a scale of 1 to 5 (grade 1, minimal; grade 5, severe) by a board-certified pathologist (Experimental Pathology Laboratories® [Sterling, VA]). The results of the histopathological evaluation have been previously published [19].

#### Metabolomics

Metabolomic analysis was conducted by Metabolon (Durham, NC). Briefly, frozen plasma samples (150 μl) were thawed, and extracts were prepared according to Metabolon’s standard protocol, which is designed to remove protein, dislodge small molecules bound to protein or physically trapped in the precipitated protein matrix and recover a wide range of chemically diverse metabolites. Samples were extracted and split into equal parts for analysis on the gas chromatography mass spectrometer and liquid chromatography mass spectrometer platforms. Proprietary software was used to match ions to an in-house library of standards for metabolite identification and for metabolite quantitation by peak area integration.

#### Data analysis—metabolomics

Two-way ANOVA with contrasts was used to analyze the data (See Additional file 1). For all analyses, missing values (if any) were inputted with the observed minimum for that particular compound (inputted values were added after block-normalization). The statistical analyses were performed on natural log-transformed data to reduce the effect of any potential outliers in the data. Two-way ANOVA and contrast comparisons were made between the means of each biochemical from the groups and were calculated using either or both of the statistical analysis software programs Array Studio (Omicsoft, Inc.) or ‘R’ (R Foundation for Statistical Computing, Vienna, Austria). Statistical cut-offs are typically used to detemine physiological significance in metabolomics studies. Conservative criteria of p < 0.05 and q < 0.1 are routinely used in metabolomic studies [61], allowing for the identification of significantly altered responses between groups with a false discovery rate of no more than 10%. Because we analyzed a constellation of metabolites in conjunction with biochemical pathways rather than single analytes, we considered p < 0.05 significant, regardless of the q value. Using this approach, we could be more inclusive of data that did not meet strict cut-off values, taking both p-value and number of metabolites changing in a given biochemical pathway.

Random forest, a supervised classification technique based on an ensemble of decision trees [62,63] was used to determine the predictive value of multiple biochemicals for both exposure and health effect. For a given decision tree, a random subset of the data with identifying true class information was selected to build the tree (“bootstrap sample” or “training set”), and then the remaining data, the OOB variables, were passed down the tree to obtain a class prediction for each sample, then repeated thousands of times to produce the forest. The final classification of each sample was determined by computing the class prediction frequency for the OOB variables over the whole forest. Class predictions were compared to the true classes, generating the “OOB error rate” as a measure of prediction accuracy. To determine which variables (biochemicals) made the largest contribution to the classification, a variable importance measure was computed, termed the mean decrease accuracy (MDA). The MDA was determined by randomly permuting a variable, running the observed values through the trees, and then reassessing the prediction accuracy. If a variable is not important, then this procedure had little change in the accuracy of the class prediction (permuting random noise gave random noise). By contrast, if a variable is important to the classification, the prediction accuracy will drop after such a permutation, which we record as the MDA. Thus, the random forest analysis provided an importance rank-ordering of biochemical, and the top 30 biochemicals were reported.

## Additional files

Additional file 1:
**Is the raw metabolomics data and ANOVA with contrasts for each biochemical analyzed in the study.**
Additional file 2:
**Heat stress persistently increases metabolites controlling pyrimidine and purine degradation.** **(A)** Eighteen biochemicals were identified in the purine and pyrimidine metabolism sub-pathways: at T_c,Max_, fold changes (heat/control) were significantly increased for 12 biochemicals and significantly decreased for 1 biochemical; at 24 hours, 8 biochemicals were increased and 2 decreased; and at 48 hours, 4 were elevated and 2 decreased in both uninjured and heat-injured animals. **(B)** Biochemical pathway for pyrimidine degradation. Red, increased over control; green, decreased relative to control; green and red, p < 0.05; light red, 0.05 < p < 0.1. Additional file 3:
**Acute increase in acetylated biochemicals followed by a decrease at 24 hours and significant differences between uninjured animals and animals with cardiac injury at 48 hours.** Of the 13 acetylated biochemicals at T_c,Max_, 12 were greater in heated animals than controls; at 24 hours, 5 were significantly lower and 2 were significantly higher; at 48 hours in uninjured animals, 3 were significantly higher and 2 were significantly lower; in heat-injured animals, 3 were higher and 5 were lower than controls. Red, fold change significantly higher; green, fold change significantly lower than control (p < 0.05 by ANOVA); light green, fold change lower compared to controls (0.05 < p < 0.1 by ANOVA). Additional file 4:
**Acute increase in sulfated biochemicals followed by a decrease at 24 hours and significant differences between uninjured animals and animals with cardiac injury at 48 hours.** Of the 8 sulfated biochemicals at T_c,Max_, 7 were greater in heated animals than controls; at 24 hours, 4 were significantly lower and none were significantly higher; at 48 hours in uninjured animals, 3 were significantly higher and none were significantly lower; and in heat-injured animals, 4 were higher and none were lower than controls. Red, fold change significantly higher; green, fold change significantly lower than control (p < 0.05 by ANOVA); light green, fold change slightly lower compared to controls with 0.05 < p < 0.1 by ANOVA. Additional file 5:
**Altered amino acids and mediators of glycolysis, gluconeogenesis, and pyruvate metabolism after heat stress and recovery.** **(A)** Of the 19 identified amino acids, at T_c,Max_ 7 were significantly lower and 8 were significantly greater; 4 were lower at 24 hours; at 48 hours, 5 were lower and 1 was greater in both uninjured and cardiac-injured animals. Metabolites contributing to glycolysis were initially greater than control, then either returned to normal or were lower than control at 24 and 48 hours. Red, fold change significantly higher; green, fold change significantly lower than control (p < 0.05 by ANOVA); light green, fold change slightly lower compared to controls; light red, fold change lightly higher than control (0.05 < p < 0.1 by ANOVA). **(B)** Three amino acids that contribute to the tricarboxylic acid (TCA) cycle for energy production were decreased. Additional file 6:
**Increased fatty acid β-Oxidation modulates acetyl CoA production and cholesterol synthesis in energy production.** **(A)** Metabolic network illustrating how elevations in fatty acid β-oxidation compensates for compromised TCA cycle, glycolysis, and amino acid metabolism in the production of cellular energy. **(B)** Alterations in medium chain fatty acids in response to heat stress and recovery. **(C)** Alterations in biochemicals involved in carnitine metabolism after heat stress. Green cells represent significant decrease. Red cells represent a significant increase in heat-stressed individuals over control cases (0.05 < *p* < 0.10 heat exposed versus control rat, 2-way ANOVA with contrasts). Additional file 7:
**Decrease in bile acids and increase in associated metabolites of bile acid biosynthesis at T**_**c,Max**_ **and 24–48 hours after heat exposure.** **(A)** Tabulation of bile acids and associated metabolites at T_c,Max_ and 24–48 hours as fold-change from control after heat exposure. **(B)** Circulation of bile acids. **(C)** Trend in bile acid and associated metabolites over time after heat stress. Green cells represent significant decrease. Pink reflects a trending increase and light green represents a trending decrease (0.05 < *p* < 0.10 heat exposed versus control rat, 2-way ANOVA with contrasts); *, p < 0.05, 2-way ANOVA with contrasts. Additional file 8:
**Change in vita** **min B6 cofactor activity at T**_**c,Max**_ **and 24–48 hours after heat exposure.** **(A)** Role of vitamin B6 metabolism in amino acid, serotonin/norepinephrine, and sphingolipid synthesis. Trend in **(B)** pyridoxal and **(C)** pyridoxate after heat stress. *, p < 0.05, 2-way ANOVA with contrasts.

## Abbreviations

7-HOCA: 7-α-hydroxy-3-oxo-4-cholestenoate
AA: Amino acid
acetyl CoA: Acetyl coenzyme A
AHB: 2-hydroxybutyrate
AMP: Adenosine monophosphate
ANOVA: Analysis of variance
ATP: Adenosine triphosphate
GCS: γ-glutamyl cysteine synthase
GGT: γ -glutamyl transferase
GSH: Glutathione
GS: Glutathione synthase
GSSG: Glutathione disulfide, oxidized
KEGG: Kyoto encyclopedia of genes and genomes
MDA: Mean decrease accuracy
NADPH: Nicotinamide adenine dinucleotide phosphate
NO: Nitric oxide
OOB: Out-of-bag
ROS: Reactive oxygenated species
SIRS: System inflammatory response syndrome
T_c,Max_: Maximum core temperature
TCA: Tricarboxylic acid
USACEHR: US Army Center for Environmental Health Research
USAMRMC: US Army Medical Research and Materiel Command
USARIEM: US Army Research Institute of Environmental Medicine
