# Supplementary material for: Alteration in circulating metabolites during and after heat stress in the conscious rat: potential biomarkers of exposure and organ-specific injury
Source: BMC Physiol. 2014 Dec 24;14:14. doi: 10.1186/s12899-014-0014-0 (PMC4306243; doi:10.1186/s12899-014-0014-0)
Supplement: Additional file 2: — Heat stress persistently increases metabolites controlling pyrimidine and purine degradation. (A) Eighteen biochemicals were identified in the purine and pyrimidine metabolism sub-pathways: at Tc,Max, fold changes (heat/control) were significantly increased for 12 biochemicals and significantly decreased for 1 biochemical; at 24 hours, 8 biochemicals were increased and 2 decreased; and at 48 hours, 4 were elevated and 2 decreased in both uninjured and heat-injured animals. (B) Biochemical pathway for pyrimidine degradation. Red, increased over control; green, decreased relative to control; green and red, p < 0.05; light red, 0.05 < p < 0.1. [file 12899_2014_14_MOESM2_ESM.pdf]

A

Biochemical Name	Fold change, Heat/Control			
	$T_{c,Max}$	24 hr Recovery	Heat 48 hr Uninjured	Heat 48 hr Injured
**Purine Metabolism**				
adenosine 5'-monophosphate (AMP)	-1.33	-2.08	1.24	1.34
N1-methyladenosine	1.68	1.37	1.27	-1.10
N6-carbamoylthreonyladenosine	2.92	1.33	1.35	1.43
allantoin	6.30	1.04	1.21	-1.01
urate	5.20	1.11	1.11	1.02
**Pyrimidine Metabolism**				
2'-deoxycytidine	1.19	1.22	1.08	1.05
5-methyl-2'-deoxycytidine	1.21	1.10	1.41	1.30
5-methylcytidine	1.21	1.10	1.05	1.06
cytidine	-1.19	1.22	-1.04	-1.03
cytosine	1.14	1.22	-1.20	-1.09
N4-acetylcytidine	1.61	-1.06	1.61	1.87
dihydroorotate	-1.03	-1.43	-1.25	-1.16
orotate	1.51	-1.03	-1.03	-1.06
thymidine	1.85	1.81	1.05	1.07
5,6-dihydrouracil	1.32	-1.37	-1.34	-1.87
pseudouridine	1.80	1.24	1.35	1.16
uracil	1.45	1.28	1.14	1.21
uridine	-1.06	1.02	-1.28	-1.15

B

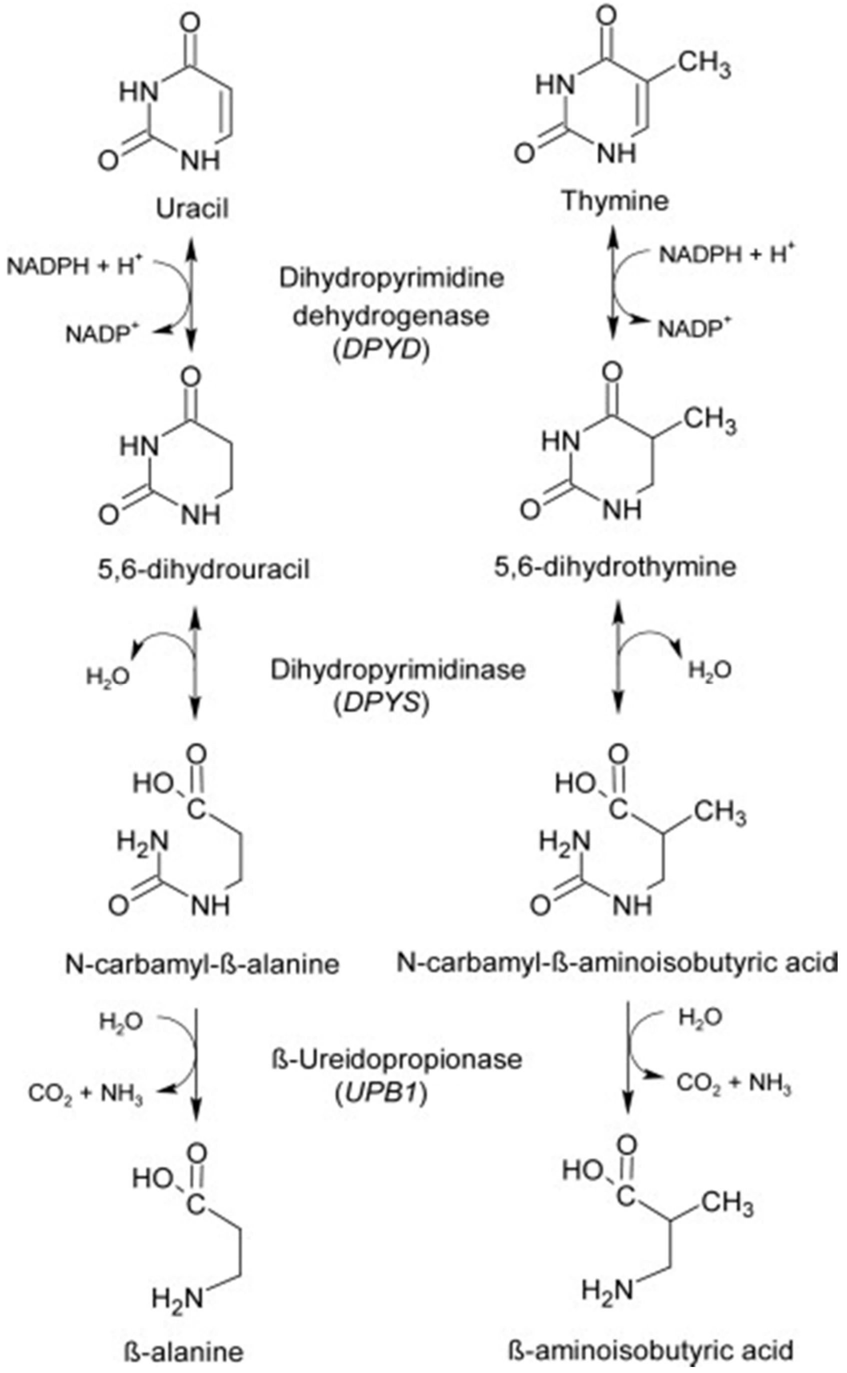

Additional File 2
