# Supplementary material for: Alteration in circulating metabolites during and after heat stress in the conscious rat: potential biomarkers of exposure and organ-specific injury
Source: BMC Physiol. 2014 Dec 24;14:14. doi: 10.1186/s12899-014-0014-0 (PMC4306243; doi:10.1186/s12899-014-0014-0)
Supplement: Additional file 3: — Acute increase in acetylated biochemicals followed by a decrease at 24 hours and significant differences between uninjured animals and animals with cardiac injury at 48 hours. Of the 13 acetylated biochemicals at Tc,Max, 12 were greater in heated animals than controls; at 24 hours, 5 were significantly lower and 2 were significantly higher; at 48 hours in uninjured animals, 3 were significantly higher and 2 were significantly lower; in heat-injured animals, 3 were higher and 5 were lower than controls. Red, fold change significantly higher; green, fold change significantly lower than control (p < 0.05 by ANOVA); light green, fold change lower compared to controls (0.05 < p < 0.1 by ANOVA). [file 12899_2014_14_MOESM3_ESM.pdf]

Additional File 3

	Fold change, Heat/Control			
	$T_{c,Max}$	24 hr Recovery	Heat 48 hr Uninjured	Heat 48 hr Injured
N-acetylalanine	**1.99**	1.32	-1.01	-1.54
N-acetyl-beta-alanine	**2.70**	**1.72**	**1.29**	**1.31**
N-acetylglycine	**1.37**	1.02	1.02	1.23
N-acetylisoleucine	**5.38**	**-1.35**	1.04	**-1.54**
N-acetylleucine	**4.81**	**-1.39**	1.30	-1.20
N-acetylmethionine	**3.78**	**-1.32**	1.29	-1.03
N-acetylneuraminate	**2.62**	**1.59**	**1.45**	1.17
N-acetylornithine	-1.01	**-1.32**	-1.18	**-1.50**
N-acetylphenylalanine	**1.96**	-1.33	1.25	1.02
N-acetylthreonine	**3.10**	1.08	**1.65**	**1.35**
N-acetyltryptophan	**4.18**	**-1.49**	1.31	-1.06
N-acetyltyrosine	**2.76**	**-1.96**	-1.25	**-1.84**
N-acetylvaline	**4.34**	1.00	1.00	1.00
