# Supplementary material for: Alteration in circulating metabolites during and after heat stress in the conscious rat: potential biomarkers of exposure and organ-specific injury
Source: BMC Physiol. 2014 Dec 24;14:14. doi: 10.1186/s12899-014-0014-0 (PMC4306243; doi:10.1186/s12899-014-0014-0)
Supplement: Additional file 4: — Acute increase in sulfated biochemicals followed by a decrease at 24 hours and significant differences between uninjured animals and animals with cardiac injury at 48 hours. Of the 8 sulfated biochemicals at Tc,Max, 7 were greater in heated animals than controls; at 24 hours, 4 were significantly lower and none were significantly higher; at 48 hours in uninjured animals, 3 were significantly higher and none were significantly lower; and in heat-injured animals, 4 were higher and none were lower than controls. Red, fold change significantly higher; green, fold change significantly lower than control (p < 0.05 by ANOVA); light green, fold change slightly lower compared to controls with 0.05 < p < 0.1 by ANOVA. [file 12899_2014_14_MOESM4_ESM.pdf]

Additional File 4

	Fold change, Heat/Control			
**Biochemical Name**	$T_{c,Max}$	**24 hr Recovery**	**Heat 48 hr Uninjured**	**Heat 48 hr Injured**
cysteine	**1.96**	-1.03	1.25	1.05
p-cresol sulfate	**1.66**	-1.02	1.33	1.37
phenol sulfate	**3.54**	**-1.61**	-1.06	**-1.28**
3-indoxyl sulfate	**2.84**	-1.14	-1.10	1.03
4-ethylphenylsulfate	1.16	**-1.54**	**1.37**	1.05
4-vinylphenol sulfate	**2.78**	**-2.00**	**1.79**	1.08
catechol sulfate	**1.40**	-1.15	**1.46**	1.20
4-acetylphenol sulfate	**5.23**	**-1.69**	**1.77**	1.29
