# Supplementary material for: Alteration in circulating metabolites during and after heat stress in the conscious rat: potential biomarkers of exposure and organ-specific injury
Source: BMC Physiol. 2014 Dec 24;14:14. doi: 10.1186/s12899-014-0014-0 (PMC4306243; doi:10.1186/s12899-014-0014-0)

Additional File 5

A

Biochemical Name	Fold change, Heat/Control			
	$T_{c,Max}$	24 hr Recovery	Heat 48 hr Uninjured	Heat 48 hr Injured
**Amino Acids**				
alanine	-1.69	-1.16	-1.18	-1.09
proline	-1.56	-1.10	-1.18	-1.17
arginine	-1.41	-1.04	-1.05	1.05
asparagine	-1.35	-1.33	-1.22	-1.25
serine	-1.32	-1.11	-1.15	1.11
threonine	-1.32	-1.06	-1.14	-1.11
histidine	-1.14	-1.03	-1.04	1.02
glycine	-1.12	1.01	1.02	1.09
glutamine	1.06	1.05	1.01	1.15
phenylalanine	1.06	1.02	-1.03	1.07
tyrosine	1.14	1.06	1.01	1.10
methionine	1.15	-1.03	1.03	1.08
isoleucine	1.18	-1.14	-1.11	-1.08
lysine	1.22	1.62	1.60	2.87
leucine	1.25	-1.08	-1.14	-1.10
valine	1.27	-1.15	-1.14	-1.08
tryptophan	1.40	-1.18	-1.04	-1.02
cysteine	1.96	-1.03	1.05	1.25
**Glycolysis, gluconeogenesis, pyruvate metabolism**				
glutamate	2.87	-1.45	-1.34	-1.16
1,3-dihydroxyacetone	-1.47	1.09	1.18	-1.04
glucose	-1.11	-1.15	-1.08	-1.00
pyruvate	-1.09	-1.20	1.01	-1.25
lactate	1.03	-1.05	-1.15	-1.10
1,5-anhydroglucitol (1,5-AG)	1.20	-1.11	-1.01	1.01
3-phosphoglycerate	1.37	1.05	1.36	1.38
glycerate	1.56	-1.25	-1.41	-1.99

B

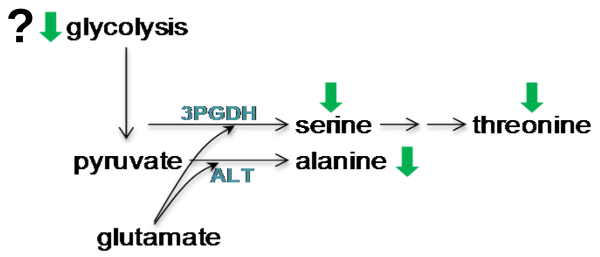

Supplement: Additional file 5: — Altered amino acids and mediators of glycolysis, gluconeogenesis, and pyruvate metabolism after heat stress and recovery. (A) Of the 19 identified amino acids, at Tc,Max 7 were significantly lower and 8 were significantly greater; 4 were lower at 24 hours; at 48 hours, 5 were lower and 1 was greater in both uninjured and cardiac-injured animals. Metabolites contributing to glycolysis were initially greater than control, then either returned to normal or were lower than control at 24 and 48 hours. Red, fold change significantly higher; green, fold change significantly lower than control (p < 0.05 by ANOVA); light green, fold change slightly lower compared to controls; light red, fold change lightly higher than control (0.05 < p < 0.1 by ANOVA). (B) Three amino acids that contribute to the tricarboxylic acid (TCA) cycle for energy production were decreased. [file 12899_2014_14_MOESM5_ESM.pdf]
