# Supplementary material for: Alteration in circulating metabolites during and after heat stress in the conscious rat: potential biomarkers of exposure and organ-specific injury
Source: BMC Physiol. 2014 Dec 24;14:14. doi: 10.1186/s12899-014-0014-0 (PMC4306243; doi:10.1186/s12899-014-0014-0)
Supplement: Additional file 6: — Increased fatty acid β-Oxidation modulates acetyl CoA production and cholesterol synthesis in energy production. (A) Metabolic network illustrating how elevations in fatty acid β-oxidation compensates for compromised TCA cycle, glycolysis, and amino acid metabolism in the production of cellular energy. (B) Alterations in medium chain fatty acids in response to heat stress and recovery. (C) Alterations in biochemicals involved in carnitine metabolism after heat stress. Green cells represent significant decrease. Red cells represent a significant increase in heat-stressed individuals over control cases (0.05 < p < 0.10 heat exposed versus control rat, 2-way ANOVA with contrasts). [file 12899_2014_14_MOESM6_ESM.pdf]

Additional File 6

A

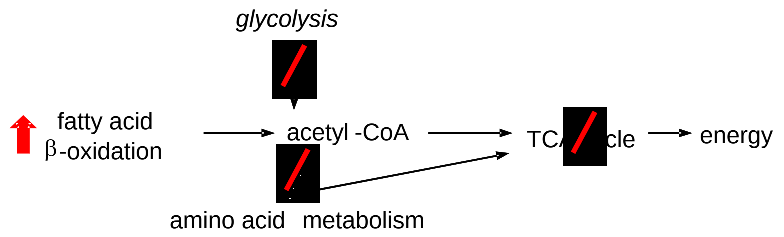

B

Biochemical Name	Fold change, Heat/Control			
	$T_{c,Max}$	24 hr Recovery	Heat 48 hr Uninjured	Heat 48 hr Injured
pelargonate (9:0)	**-1.96**	1.02	1.08	-1.09
heptanoate (7:0)	**-1.85**	-1.05	-1.01	-1.32
caprate (10:0)	**-1.43**	1.01	1.15	1.08
caprylate (8:0)	**-1.33**	-1.03	1.06	-1.12
undecanoate (11:0)	-1.27	**1.52**	-1.09	1.11
laurate (12:0)	-1.18	1.12	1.08	1.11
caproate (6:0)	-1.03	-1.05	-1.05	-1.02
isocaproate	**2.59**	**2.26**	1.21	1.61

C

Biochemical Name	Fold change, Heat/Control			
	$T_{c,Max}$	24 hr Recovery	Heat 48 hr Uninjured	Heat 48 hr Injured
3-dehydrocarnitine*	1.17	1.16	-1.17	-1.27
acetylcarnitine	**1.24**	1.04	-1.01	1.07
carnitine	1.03	1.11	-1.06	1.21
deoxycarnitine	-1.12	1.08	-1.14	-1.04
hexanoylcarnitine	**1.41**	1.28	**1.35**	1.58
oleoylcarnitine	-1.05	1.52	**2.67**	3.17
palmitoylcarnitine	-1.22	1.10	**2.37**	2.67
stearoylcarnitine	-1.30	1.07	**1.87**	1.98
3-hydroxybutyrate (BHBA)	**2.58**	-1.10	-1.00	-1.11
