# Supplementary material for: Alteration in circulating metabolites during and after heat stress in the conscious rat: potential biomarkers of exposure and organ-specific injury
Source: BMC Physiol. 2014 Dec 24;14:14. doi: 10.1186/s12899-014-0014-0 (PMC4306243; doi:10.1186/s12899-014-0014-0)
Supplement: Additional file 7: — Decrease in bile acids and increase in associated metabolites of bile acid biosynthesis at T c,Max and 24–48 hours after heat exposure. (A) Tabulation of bile acids and associated metabolites at Tc,Max and 24–48 hours as fold-change from control after heat exposure. (B) Circulation of bile acids. (C) Trend in bile acid and associated metabolites over time after heat stress. Green cells represent significant decrease. Pink reflects a trending increase and light green represents a trending decrease (0.05 < p < 0.10 heat exposed versus control rat, 2-way ANOVA with contrasts); *, p < 0.05, 2-way ANOVA with contrasts. [file 12899_2014_14_MOESM7_ESM.pdf]

Additional File 7

A

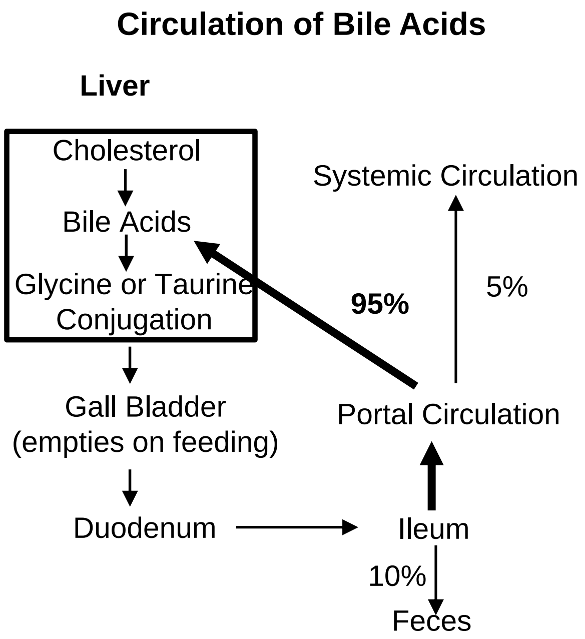

B

Biochemical Name	Fold change, Heat/Control			
	$T_{c,Max}$	24 hr Recovery	Heat 48 hr Uninjured	Heat 48 hr Injured
**Sterol/Steroid**				
7-alpha-hydroxy-3-oxo-4-cholestenoate (7-Hoca)	1.52	1.79	1.53	1.93
7-alpha-hydroxycholesterol	-1.20	2.93	3.01	2.80
beta-sitosterol	-1.04	-1.20	-1.09	-1.25
campesterol	-1.11	-1.22	-1.14	-1.12
cholesterol	-1.23	-1.01	1.06	1.00
corticosterone	3.07	2.59	1.28	1.92
**Bile Acid Metabolism**				
6-beta-hydroxylithocholate	-1.64	1.09	-1.45	1.40
beta-muricholate	-5.56	1.75	1.13	-1.09
chenodeoxycholate	-8.33	-1.11	-1.08	-1.39
cholate	-9.09	-1.19	-2.17	1.24
deoxycholate	-1.64	-1.05	-1.32	1.24
glycocholate	-3.57	1.03	1.52	1.16
taurochenodeoxycholate	-2.38	-1.79	1.36	-1.37
taurocholate	-9.09	-2.94	1.55	-1.23
taurodeoxycholate	-4.00	-1.52	-1.21	1.06
tauroursodeoxycholate	-2.13	-2.04	1.30	-1.27
