# Supplementary figures and images for: Alteration in circulating metabolites during and after heat stress in the conscious rat: potential biomarkers of exposure and organ-specific injury
Source: BMC Physiol. 2014 Dec 24;14:14. doi: 10.1186/s12899-014-0014-0 (PMC4306243; doi:10.1186/s12899-014-0014-0)

# Additional File 8

A

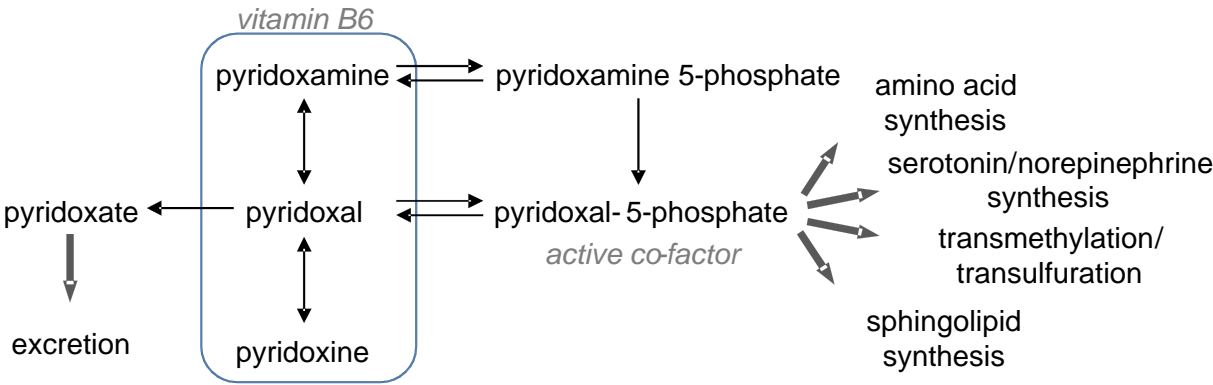

B

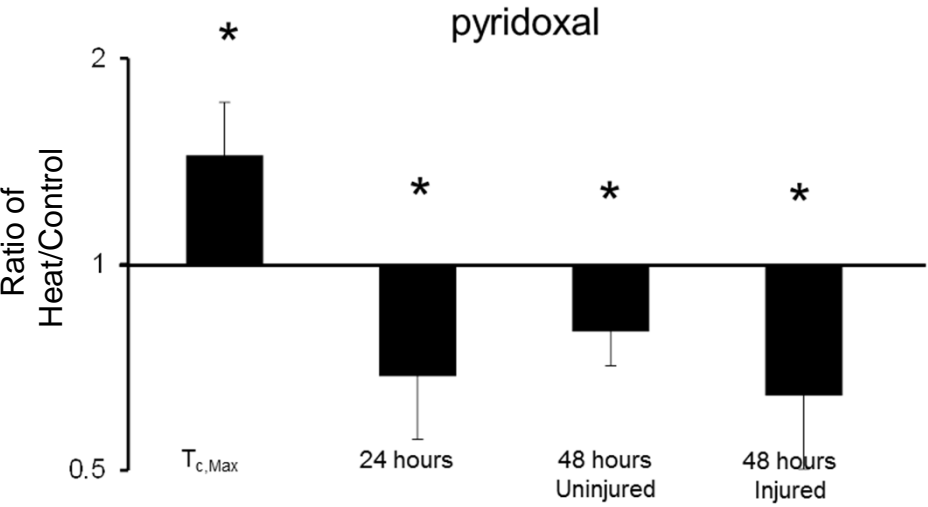

C

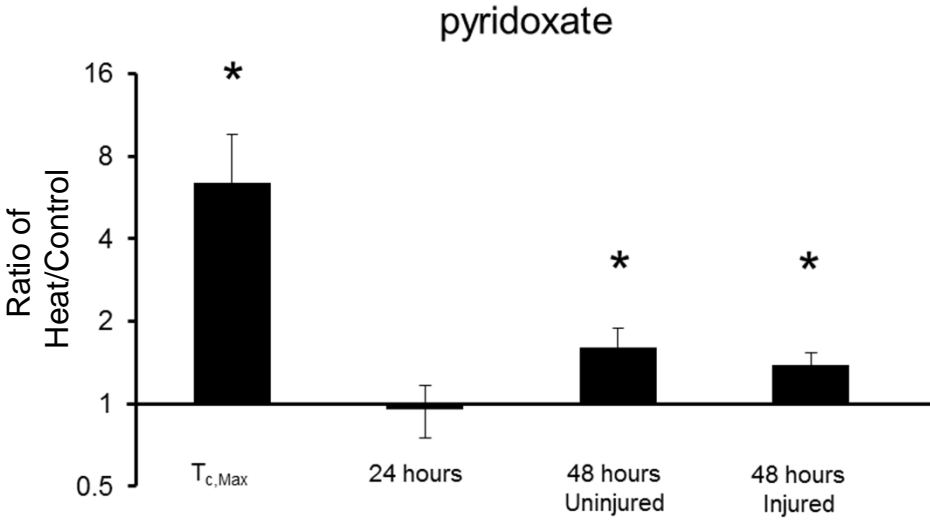

Supplement: Additional file 8: — Change in vita min B6 cofactor activity at T c,Max and 24–48 hours after heat exposure. (A) Role of vitamin B6 metabolism in amino acid, serotonin/norepinephrine, and sphingolipid synthesis. Trend in (B) pyridoxal and (C) pyridoxate after heat stress. *, p < 0.05, 2-way ANOVA with contrasts. [file 12899_2014_14_MOESM8_ESM.pdf]
